# Cardiac Morphofunctional Characteristics of Individuals with Early Repolarization Pattern: A Literature Review

**DOI:** 10.3390/jcdd10010004

**Published:** 2022-12-22

**Authors:** Loránd Kocsis, Zsuzsanna Pap, Attila Frigy

**Affiliations:** 1 Department of Cardiology, Clinical County Hospital Mures, 540103 Targu Mures, Romania; 2 Department of Anatomy and Embryology, George Emil Palade University of Medicine, Pharmacy, Science and Technology of Targu Mures, 540103 Targu Mures, Romania; 3 Department of Internal Medicine IV, George Emil Palade University of Medicine, Pharmacy, Sciences and Technology of Targu Mures, 540103 Targu Mures, Romania

**Keywords:** early repolarization pattern, morphofunctional changes, echocardiography, speckle tracking echocardiography, magnetic resonance imaging

## Abstract

The early repolarization pattern (ERP) is an electrocardiographic phenomenon characterized by the appearance of a distinct J-wave or J-point elevation at the terminal part of the QRS complex. ERP is associated with an increased risk of ventricular arrhythmias in susceptible individuals. The cardiac morphofunctional parameters in subjects with ERP have been characterized mainly by imaging techniques, which suggests that certain changes could be identified in the background of the electrical pathomechanism: however, in this regard, current data are often contradictory or insufficiently detailed. For clarification, a more comprehensive cardiac imaging evaluation of a large patient population is necessary. This review summarizes and analyses the data from the literature related to cardiac morphofunctional characteristics in individuals with ERP.

## 1. Introduction

**Figure 1:**
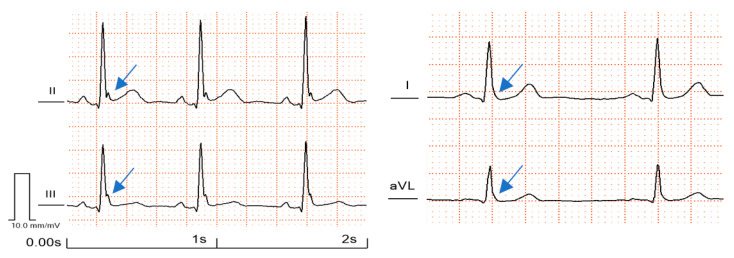
Different manifestations and localization of J-waves on the surface ECG. ERP with notching morphology in the inferior leads (on the left side). ERP with slurring morphology in the lateral leads (on the right side). The J-waves are marked with arrows. ECGs from the authors’ own collection.

**Figure 2:**
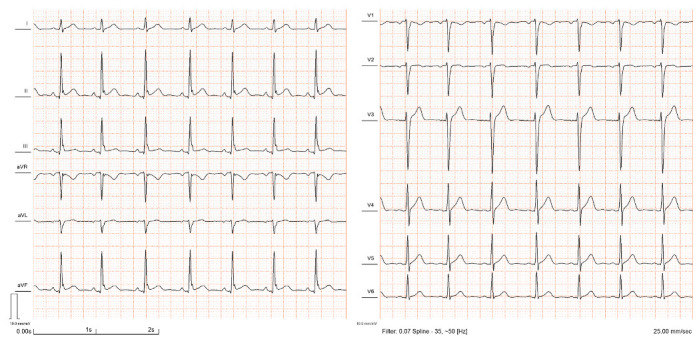
ECG registration with ERP with notching morphology in the inferior leads. The ECG is from the authors’ personal collection.

The early repolarization pattern (ERP) is an electrocardiographic (ECG) phenomenon characterized by a distinct J-wave or J-point elevation at the terminal part of the QRS (Figure 1 and Figure 2): this notch- or slur-like abnormality, which may be accompanied by ST segment elevation, has traditionally been considered a benign entity [1,2,3].

The presence of J-waves on the ECG, and their possible association with life-threatening cardiac arrhythmias or sudden cardiac death (SCD), has been suspected for a long time, and now been confirmed by clinical and experimental data, including case reports describing patients with hypothermia, hypercalcemia, and ischemia [4,5,6]. At the turn of the millennium, the association between the presence of ERP and ventricular tachycardia (VT) or ventricular fibrillation (VF) was demonstrated by experimental data obtained from canine ventricular wedge preparations [3,7]. Several observational studies have shown that the presence of ERP in the inferior and/or lateral leads is more frequently observed on the 12-lead ECG of resuscitated patients. Subsequently, further clinical studies have confirmed that there is an association between ERP and malignant ventricular arrhythmias or SCD. In addition, the presence of ERP has been shown to increase the likelihood of future malignant arrhythmias [8,9,10].

If ERP is associated with VT or VF, and there exists no demonstrable cardiac disease, it is referred to as early repolarization syndrome (ERS). According to some researchers, benign and malignant ERP can be distinguished from one another by their ECG appearance [11,12]. ERS has several clinical similarities to Brugada syndrome (BrS): therefore, according to the latest consensus reports, they can be regarded as two forms of J-wave syndrome. On a surface ECG, the J-waves appear in leads V1-V3 in BrS, whereas they appear in the inferior and/or lateral leads in ERS [2].

## 2. Diagnostic Criteria

The latest definitions, diagnostic criteria, and measurement recommendations of ERP are detailed in a consensus document published by Macfarlane et al. in 2015 [13].

According to the consensus document, the leads showing the ERP should be mentioned in the description of the pattern; furthermore, it is recommended that the ST segment slope be defined when describing the pattern, as a horizontal or downward sloping ST segment has a worse prognosis than an ascending one [14,15]. The diagnostic criteria for ERP are presented in Table 1.

Although the diagnostic criteria for ERP are well-defined, a scientific statement from the American Heart Association published in 2016 highlights the discrepancies used in describing the pattern in scientific publications [16]. In the 2017 international consensus document on J-wave syndrome, a diagnostic scoring system (Shanghai ERS Score) was proposed for the correct diagnosis of ERS, based on the latest evidence in the literature [2].

## 3. Prevalence

The prevalence of ERP in the inferior and/or lateral leads ranges from 1% to 31% in the general population, and from 15% to 70% in individuals with idiopathic VF [17,18,19].

ECGs showing ERP are more commonly observed in young people. More than 70% of individuals with ERP are men, and the difference between the sexes decreases with age, suggesting a hormonal effect (related to testosterone) [20,21]. ERP is more common in young athletes, where it occurs in 31% of subjects [14].

According to some studies, ERP occurs at a slightly higher rate in African Americans, Southeast Asians, and Australian Aborigines; however, another study found that there is no correlation between the occurrence of ERP in different geographic regions [15,19,22].

It is important to note that ERP can be associated with several factors that predispose to arrhythmias, such as high vagal tone, hypothermia, bradycardia, prolonged QRS, short QT interval, left ventricular hypertrophy (e.g., based on Sokolow–Lyon index), and fragmented QRS [12,23,24].

## 4. Arrhythmic Risk

Several studies have estimated the risk of SCD in the ERP population. According to a Finnish study that examined more than 10,000 individuals, a correlation was found between a J-point elevation of at least 0.1 mV in the inferior or lateral leads and arrhythmia-related cardiac death [17]; however, in an American study with 29,000 participants, no association was found between the degree of J-point elevation and cardiovascular mortality [16]. As reported by Rosso et al., in a study of 100,000 individuals between 35 and 45 years of age, the presence of a J-wave increased the probability of VF from 3.4 to 11 [9,12]. According to Arroyo et al., in a small number of cases ERP was the only cause of SCD. Arroyo et al. also suggested the role of ERP as a disease-modifying factor rather than an independent substrate [25].

Antzelovitch et al. described three forms of ERP, based on ECG localization, with different degrees of VF risk. ERP occurring in the lateral ECG leads is common in healthy young male athletes but is rarely observed in VF survivors, and is therefore considered to carry a very low risk of arrhythmia (type 1). ERP observed in the inferior or infero-lateral leads is most seen in young males and in individuals who have experienced VF, and thus carries a higher risk of arrhythmia (type 2). The rarely occurring global (infero-lateral + right praecordial) ERP is considered to carry the highest VF risk (type 3) [1,2,3,7].

According to Tikkanen et al., the amplitude of J-point elevation is also associated with mortality caused by cardiac arrhythmias: of the population that they studied, a J-point elevation of ≥0.1 mV was present in 5.8%, whereas a J-point elevation of ≥0.2 mV was present in only 0.3%. Tikkanen et al. demonstrated a correlation between a J-point elevation of ≥0.1 mV in the inferior leads and cardiovascular mortality (RR = 1.28)/death due to arrhythmias (RR = 1.43); for a J-point elevation of ≥0.2 mV, they identified a higher risk of cardiovascular mortality and arrhythmia-related death (RR = 2.92 in both cases) [14,17]. These results were confirmed later by several other studies of subjects who died from different cardiovascular diseases [18,19,21]. An electrophysiological study by Mahida et al. showed no relationship between J-wave amplitude and VF in patients with ERS; however, the sample size of this study was relatively small, and the authors used programmed stimulation for the induction of VF [22].

With regard to ERP morphology, it has been found that the risk of ventricular arrhythmias is the same between the two ERP subgroups—the slur and the notch J-wave patterns [14], while several studies have shown that a horizontal or descending ST segment slope following the J-wave is associated with a worse prognosis than an ascending ST segment [14,15].

Recently published studies have shown that ERP is associated with an increased risk of atrial fibrillation [26], while structural and functional remodeling of the left atrium can be characterized with modern imaging techniques, such as 3D speckle tracking echocardiography [27]. As the degree of atrial fibrosis correlates with the appearance of arrhythmias, it would be useful to evaluate the atrial fibrosis in individuals with ERP using 3D speckle tracking echocardiography or other imaging modalities.

To summarize the results of the published scientific works, the risk of fatal arrhythmia is higher in individuals whose ECG shows a J-wave of ≥0.2 mV in the inferior or infero-lateral lead, followed by a horizontal or descending ST segment.

As a therapeutical approach, ICD implantation is recommended in patients who have survived a cardiac arrest (Class I). ICD implantation or quinidine may be considered in individuals with arrhythmic syncope and additional risk features, and in asymptomatic individuals who demonstrate a high-risk ERP in the presence of a family history of unexplained juvenile sudden death (Class IIb). Isoproterenol infusion could be helpful in ERS patients with electrical storm (Class IIa) [28].

## 5. Electrophysiological and Genetic Background

**Figure 3:**
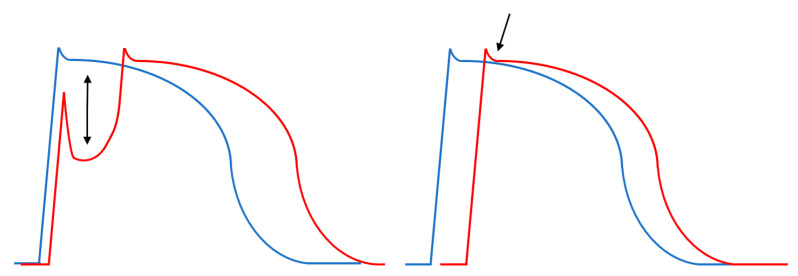
Schematic representation of J-wave genesis at the action potential level. The **left** figure shows the transmural voltage gradient, characteristic of the early repolarization theory. On the **right** is the delayed impulse conduction that occurs at the level of the epicardium. The epicardium is shown with a red line, the endocardium with a blue line. The arrows show the voltage (left) and temporal (right) differences in the background of J-wave formation.

The pathophysiological mechanism of J-wave genesis is currently not fully understood. There are two theories of the origin of J-waves: the first theory considers them to be a repolarization abnormality, while the second theory considers them to be a depolarization abnormality (Figure 3).

According to a theory developed by Antzelevitch et al., an enhanced notch, compared to the endocardium, occurs in the first phase of the action potential of the epicardium: this abnormality is explained by an increase in the transient outward potassium current, occurring as a result of a genetic mutation. The differential, transmural distribution of the transient outward current leads to an increase in the transmural voltage gradient, which appears as a J-wave on the surface ECG. Subsequently, it was demonstrated that dysfunction of sodium, calcium, and potassium ATP-dependent ion channels (Table 2) can also lead to the appearance of J-waves [1,3,7].

The depolarization theory, proposed by Haissaguerre et al., states that structural changes in the background constitute the key mechanism of J-wave development: these changes delay impulse conduction at the level of the epicardium, triggering the appearance of J-waves on the surface ECG [29,30].

## 6. Cardiac Morphofunctional Characteristics of Individuals with ERP

Based on ECG, electrophysiological, epicardial, and non-invasive electroanatomical mapping studies, the inferior and/or lateral part of the left ventricle (LV) is involved in individuals with ERP [31,32,33,34,35]. In BrS, which is also a form of J-wave syndrome, structural changes were described in the outflow tract of the right ventricle (mild fibrosis, decreased connexin-43 expression, increased collagen content, or fibro-adipose infiltration), while wall motion abnormalities and mild dilatation have been noted in imaging studies [36,37,38,39,40,41]. These structural changes are more frequently observed in patients who have died of SCD [42]. It is reasonable to assume that morphological changes, presumably similar to BrS, are also present in individuals with ERP, in myocardial regions involved in pathomechanism.

Relatively few studies have characterized the cardiac morphological and functional parameters of individuals with ERP: the results of those studies are summarized and discussed below.

### 6.1. Conventional Echocardiography

Echocardiography (ECHO) represents the most common non-invasive imaging technique that provides useful information about the structure and function of the heart: it is highly sensitive and specific in regard to the diagnosis of structural heart diseases and possible substrates for SCD; it can be considered the first-line imaging technique for the primary prevention of SCD [43]. Certain cardiac parameters differ depending on age, gender, and body surface area (BSA), even in the absence of heart disease [44,45,46]: it is important to consider this fact when planning the methodology and interpreting the results of any morphological study.

Quattrini et al. investigated the cardiac characteristics associated with ERP in elite athletes from the Italian national team, during their training for the Olympic Games: by analyzing the ECHO parameters, they found that left ventricular end-diastolic diameter (LVEDD), left ventricular posterior wall thickness (LVPWT), and left ventricular mass (LVM) were greater in individuals with ERP; there was no significant difference between the individuals with ERP (the ERP+ group) and those without ERP (the ERP- group), in regard to the other structural and functional parameters [47].

Reinhard et al. studied the ECHO characteristics related to ERP in healthy athletes, for 6 years. The age of the population was 21 ± 5 years, and ERP was present in 17% of cases. The individuals with ERP+ were predominantly male. The study found that ERP was not associated with the type of sport performed; however, a trend was observed between the prevalence of ERP and the dynamics of the sport. Among the ECHO parameters, in the ERP+ group, the relative heart volume was higher in males, while the LVPWT proved to be greater in the female population. All the other studied ECHO parameters were similar in the two groups. Left ventricular geometry (normal shape, concentric remodeling, concentric hypertrophy, eccentric hypertrophy) also occurred in the same proportion in the two groups. The correlation between high-risk subgroups of ERP (inferior type, J wave > 2 mV, notching type, or horizontal/descending ST slope) and ECHO parameters were also examined. Notching-type ERP was associated with greater left atrial diameter (LAD), LVM, and relative heart volume [48].

In 2015, Serra-Grim et al. published the results of a retrospective study, covering nearly 40 years, that followed the parameters of 299 professional football players. The study analyzed the clinical and ECHO parameters associated with ERP: the percentage of ERP+ individuals was 34.1%; the ejection fraction (EF), LVPWT, interventricular septum (IVS), and LAD were similar between the ERP+ and ERP- individuals; only the LVEDD differed, being significantly higher in the ERP+ group [49].

A Swedish study investigated the associations between ERP and cardiac structure and function, as well as the response of ERP to strenuous exercise. The participants were men over the age of 45, doing cross-country running. A total of 151 individuals participated in the study, of which 67 were in the ERP+ group. All the subjects underwent a detailed pre- and post-race cardiological examination, including transthoracic ECHO. The study analyzed relatively few ECHO parameters, and found only one parameter that differed between the two groups: the ratio (E/A) between peak early diastolic transmitral flow velocity (E) and peak late diastolic transmitral flow velocity (A), which was higher in the ERP+ group before the exercise. During the post-run examination, this difference between the groups was not detectable [50].

In a retrospective study, Miragoli et al. analyzed the data of young, healthy athletes who underwent routine cardiological examination at the University Hospital of Parma between 2006 and 2017. Data from 414 subjects, aged 12 to 17 years, were processed. The participants’ electrocardiograms and ECHO data were also collected. The results showed no significant differences between the groups, when the left ventricular diameters and volumes were examined. At the same time, in the ERP+ group, the degree of left ventricular hypertrophy was more pronounced, as evidenced by greater IVS, LVPWT, and LVM. In addition, the relative wall thickness (RWT) and LVM/BSA ratio were also higher. These results suggest concentric remodeling, so ERP was considered a possible marker for geometrical remodeling of the LV [51].

The Gutenberg Health Study was a population-based cohort study in Germany, designed as a prospective, observational, single-center study, to investigate cardiovascular risk factors. As part of the study, Trenkwalder et al. investigated the association between cardiac morphofunctional parameters and the presence of ERP, using a large number of cases (6784 men, 7094 women). The population ranged in age from 35 to 74 years, with a balanced gender ratio. The standard ECHO characteristics of ERP+ individuals were compared with those of ERP- individuals. Analysis of structural parameters in the male population revealed a significant difference in LVEDD, left ventricular end-systolic diameter (LVESD), and LVM, which were smaller in the ERP+ group. Among the markers of LV diastolic and systolic function, the E/A ratio and peak velocity of the early diastolic mitral annular motion (E’) parameters were significantly higher, and the E/E’ ratio was lower. The left ventricular end-diastolic volume (LVEDV), left ventricular end-systolic volume (LVESV), and stroke volume were also smaller. There was no significant difference between the groups in the male population, in terms of IVS, LVPWT, RWT, deceleration time, and EF parameters [52]. As with ERP+ men, ERP+ women had smaller LVEDD and LVESD, but LVM was similar between the groups. In contrast to the men, the women in the ERP+ group had greater IVS, LVPWT, and RWT. In the female population, the parameters of LV function showed significantly smaller LVEDV and stroke volume in the ERP+ group, whereas LVESV showed only a trend towards a smaller value in the ERP+ group; however, the EF, deceleration time, E’, E/A ratio, and E/E’ ratio were the same in both groups [52].

Ilkhanoff et al. investigated the relationship between ERP and ECHO parameters in the middle-aged population across 25 years, as part of the CARDIA study. From their results, individuals who maintained ERP during the research period had significantly smaller LVEDD, LVESD, and LVM. According to the authors’ interpretation, the persistence of ERP from young adulthood was related to favorable structural and functional parameters [53].

In their comprehensive study, Szabó et al. examined the echocardiographic characteristics of young men with ERP, evaluating a total of 31 parameters: in this study, the control group of the ERP+ population had similar BSA. There was a significant difference in only two parameters: the LVESV was smaller, and a mild mitral regurgitation was more common in the ERP+ group. The increased incidence of mitral regurgitation in the ERP+ group was investigated and demonstrated for the first time, although the study was performed on a relatively small number of cases [54].

According to some studies, the presence of LV false tendons is also associated with ventricular arrhythmias [55]. In their study, Liu et al. hypothesized that the presence of these fibromuscular structures might be related to the presence of ERP. According to their results, the dimensions and volumes of the left atrium, LV, and right ventricle were similar in the ERP+ and ERP- groups. The frequency of false tendons was also the same in both groups, but transverse tendons were more frequent than longitudinal tendons in the ERP+ group [56].

### 6.2. Speckle Tracking Echocardiography

Speckle tracking echocardiography (STE) is a relatively new, non-invasive imaging technique that can objectively assess global and regional myocardial function. STE can be used to determine the so-called deformation parameters (e.g., global longitudinal strain) that characterize well the mechanics of the myocardial segments. STE has a higher diagnostic value in detecting cardiac dysfunction than conventional two-dimensional (2D) ECHO [57,58].

In their study, Gülel et al. selected a control group of individuals with ERP, based on age and body mass index. In addition to 2D ECHO examination, STE was used to characterize the myocardium. Only one functional parameter differed between the two groups: the E/E’ ratio, which was smaller in the ERP+ group. No other differences were found between the groups, in regard to the standard ECHO parameters. When the LV deformation parameters were analyzed, no significant differences were found between the ERP+ and ERP- groups regarding the LV longitudinal parameters. Analysis of the circumferential deformation parameters showed that the early diastolic strain rate (SRE) at the level of the apex and the global SRE were higher in the ERP+ group. In addition, for the radial deformation parameters, peak strain and SRE differed between the groups, being lower in the ERP+ subjects [59].

Çöllüoğlu et al. compared the cardiac characteristics of 50 ERP+ and 50 ERP- healthy athletes. In addition to the 2D ECHO examination, the functional parameters of the LV were also analyzed, using STE. Right ventricular dimension, infero-lateral wall thickness, aortic root diameter, LAD, RWT, and LVESV were larger in the ERP+ group, and the tranmitral E-wave was smaller. The results of 2D STE also showed a significant difference between the two groups, with respect to several parameters. In the ERP+ group, the global longitudinal strain was smaller, and the strain rate, measured in the apical three-chamber view, was also smaller in the ERP+ group. The groups’ global circumferential strain and strain rate parameters were similar [60].

### 6.3. Cardiac Magnetic Resonance Imaging

Cardiac magnetic resonance imaging (cMRI) is a rapidly developing non-invasive imaging technique that can be used to describe the structure and function of the heart in detail. The advantage of cMRI over conventional ECHO examination is that it characterizes the structure of the heart more comprehensively, and provides unique information about myocardial scarring, viability, and mass. Many experts consider cMRI to be the method of choice for assessing myocardial function [61,62].

The Dallas Heart Study examined the relationship between ERP and LVM. More than 3000 men and women participated in the study, and the cardiac parameters were determined by cMRI. Certain factors that may affect cardiac parameters, such as blood pressure and BSA, were similar in the ERP+ and ERP- groups. In the ERP+ population, LVM was greater in both men and women. There were no differences between the groups, in regard to other parameters [63].

In a recently published study, the presence of J-point elevation in individuals with hypertrophic cardiomyopathy was shown to increase the risk of ventricular arrhythmias and SCD [64]. In addition, Azevedo et al. hypothesized that in individuals with hypertrophic cardiomyopathy, ERP+ individuals have greater LVM. In their retrospective study, Azevedo et al. reviewed the cMRI scans of 85 individuals. Larger LVEDV and LVM were observed in ERP+ patients, whereas the EF was smaller. The baseline parameters of the two groups were similar [65].

Table 3 summarizes the studies that have investigated the structural and functional parameters associated with ERP.

### 6.4. Particularities of Cardiac Morphofunctional Characteristics of Athletes with ERP

Most ECHO studies examining cardiac characteristics in athletes with ERP have produced conflicting results. As shown in Table 4, in some studies, certain cardiac morphological and functional parameters were associated with ERP+.

In the studies with significant differences, all but one parameter was increased in ERP+ individuals; however, according to other studies, there was no difference in most parameters. Higher values were found in athletes with ERP regarding RWT, RVD, and GLS. The majority of the studies that investigated these parameters considered only a small number of cases.

### 6.5. Particularities of Cardiac Morphofunctional Characteristics in the General Population with ERP

Certain differences in cardiac parameters related to the presence of ERP are present in the general population too (Table 5). Although most of the parameters that seem to be associated with ERP are increased in athletes, in the general population a decrease can be observed too. The Trenkwalder study, which considered a large number of cases, reported mostly contradictory results, compared to the studies examining athletes. In the general population, EF, A, DT, LAD, AoR, VAD, GLS, and GCS were not associated with ERP: of these, EF, A, DT, and GCS showed similar results in athletes.

## 7. Conclusions

The clinical significance of ERP has been demonstrated in several large-scale studies. Individuals with ERP on the surface ECG are at higher risk for life-threatening ventricular arrhythmias and SCD. Based on the results of several studies examining cardiac morphofunctional parameters with imaging techniques, it seems that structural changes are also present in the background of ERP. Nevertheless, in the majority of the currently available studies, the sample size was small, which may have led to type I statistical errors. Furthermore, there is a deficiency in the analysis of left ventricular function, due to the relatively few parameters evaluated. We believe that it is important to perform comprehensive studies examining the atria, right ventricle, and cardiac valves in addition to the parameters of the left ventricle, in order to evidence new ERP-related characteristics with clinical significance.

In further studies, it would be worthwhile to comparatively analyze the cardiac characteristics of individuals with ERP and high arrhythmic risk. It would be important to use advanced imaging techniques such as speckle tracking echocardiography or cardiac magnetic resonance imaging, in addition to traditional echocardiographic examination. Currently, there are few studies using these techniques to characterize the structure and function of the heart in individuals with ERP.

A large sample size, modern imaging techniques, and a more detailed investigation will offer the chance to clarify conflicting results, and to uncover as-yet unknown characteristics that may be contributing to the arrhythmogenic substrate in ERP individuals.

## Figures and Tables

**Table 1:** The diagnostic criteria for ERP formulated in the recent consensus paper [13]. For the diagnosis of ERP, all of the following criteria must be met.

A QRS terminal notch or slur is present in the descending part of the R wave. If a notch is present, it must be completely above the baseline. In the case of a slur, its starting point must be above the baseline.
The amplitude of the J-point elevation is ≥0.1 mV in at least two contiguous leads on the 12-lead ECG. Leads V1 to V3 must be excluded.
The QRS length must be <120 ms.

**Table 2:** Gene defects associated with ERP/ERS, and the corresponding changes of ion channel function [1,3].

Ion Channel	Function	Locus	Gene	Protein
I_K-ATP_	↑	12p11.23	KCNJ8	Kir6.1
I_K-ATP_	↑	12p12.1	ABCC9	SUR2A
I_Ca_	↓	12p13.3	CACNA1C,	Cav1.2
I_Ca_	↓	10p12.33	CACNB2b	Cavß2b
I_Ca_	↓	7q21.11	CACNA2D1	Cavα2δ1
I_Na_	↓	3p21	SCN5A	Nav1.5
I_Na_	↓	3p22.2	SCN10A	Nav1.8

↓ = loss of function, ↑ = gain of function

**Table 3:** Characteristics of studies investigating cardiac morphological and functional parameters in individuals with ERP, from different patient populations.

Study	Imaging Technique	n	Age, Years	Men, %	ERP, %	Parameter Difference *
**Athletes**						
Quattrini et al. [47]	ECHO	704	25 ± 5	62	14.0	3
Reinhard et al. [48]	ECHO	623	21 ± 5	60.7	17.3	5
Serra-Grim et al. [49]	ECHO	299	20 ± 6.4	66	31.4	1
Aagaard et al. [50]	ECHO	151	50.9 ± 4.9	100	44.3	1
Miragoli et al. [51]	ECHO	414	13.6 ± 1.8	72	22.0	3
Çöllüoğlu et al. [60]	ECHO, STE	100	35.0 ± 11.5	49	50	9
**General population**						
Trenkwalder et al. [52]	ECHO	13878	54.6 ± 11	48.9	6.6	12
Ilkhanoff et al. [53]	ECHO	1701	25.2 ± 3.5	41.9	-	3
Szabó et al. [54]	ECHO	62	22.5 ± 1.5	100	48.3	2
Liu et al. [56]	ECHO	77	31.6 ± 7.2	96.1	42.8	1
Gülel et al. [59]	ECHO, STE	60	25.5 ± 6.2	75	58.3	5
McNamara et al. [63]	cMRI	2753	43 ± 9.5	45.1	9.9	1
**Patients with HCM**						
Azevedo et al. [65]	cMRI	85	56 ± 15	62.3	10.5	3

ECHO—echocardiography; cMRI—cardiac magnetic resonance imaging; STE—speckle tracking echocardiography; n—population size; ERP—early repolarization pattern; HCM—hypertrophic cardiomyopathy; * number of parameters showing significant difference when comparing ERP+ and ERP- groups, including results of comparisons between different subgroups.

**Table 4:** The main cardiac morphological and functional parameters investigated in different studies performed on athletes. Differences between the ERP+ and ERP- individuals. The reference of the study is indicated in the square bracket, the M and F after the brackets denote the male and female subpopulation.

Parameter	Imaging Technique	Increased in ERP+	Decreased in ERP+	No Difference
LVM	ECHO	[47], [51]		[48]M, [48]F, [50], [60]
IVS	ECHO	[51]		[48]M, [48]F, [49], [50], [60]
LVPWT	ECHO	[47], [48]F, [51]		[48]M, [48]F, [49]
RWT	ECHO	[51], [60]		
LVEDD	ECHO	[47], [49], [60]		[48]M, [48]F, [50], [51]
LVESD	ECHO			[48]M, [48]F, [51]
LVEDV	ECHO			[51], [60]
LVESV	ECHO	[60]		[51]
EF	ECHO			[47], [49], [50], [60]
E	ECHO		[60]	[47]
A	ECHO			[47], [60]
E/A	ECHO	[50]		[48]M, [48]F, [60]
DT	ECHO			[48]M, [48]F
S’				[47], [50]
E’	ECHO			[47], [50]
A’	ECHO			[47], [50]
E/E’	ECHO			[48]M, [48]F, [60]
LAD	ECHO	[48], [60]		[47], [48]M, [48]F, [49], [60]
AoR	ECHO	[60]		[48]M, [48]F
RVD	ECHO	[60]		
GLS	STE	[60]		
GCS	STE			[60]

ECHO—echocardiography; STE—speckle tracking echocardiography; ERP+—subject with early repolarization pattern; LVM—left ventricular mass; IVS—interventricular septum; LVPWT—left ventricular posterior wall thickness; RWT—relative wall thickness; LVEDD—left ventricular end-diastolic diameter; LVESD—left ventricular end-systolic diameter; LVEDV—left ventricular end-diastolic volume; LVESV—left ventricular end-systolic volume; EF—ejection fraction; E—peak early diastolic transmitral flow velocity; A—peak late diastolic transmitral flow velocity; DT—deceleration time; S’—peak velocity of systolic mitral annular motion; E’—peak velocity of early diastolic mitral annular motion; A’—peak velocity of diastolic mitral annular motion; LAD—left atrial diameter; AoR—aortic root dimension; RVD—right ventricle diameter; GLS—global longitudinal strain; GCS—global circumferential strain.

**Table 5:** The main cardiac morphological and functional parameters investigated in different studies performed on the general population. The differences between ERP+ and ERP- individuals. The reference of the study is indicated in the square bracket, the M and F after the bracket denote the male and female subpopulation.

Parameter	Imaging Technique	Increased in ERP+	Decreased in ERP+	No Difference
LVM	ECHO, cMRI	[63]M, [63]F	[52]M, [53]	[52]F, [54], [59]
IVS	ECHO	[52]F		[52]M, [53], [54], [59]
LVPWT	ECHO	[52]F		[52]M, [53], [54], [56], [59]
RWT	ECHO	[52]F		[52]M, [56]
LVEDD	ECHO	[52]F, [53]	[52]M, [52]F	[54], [56], [59]
LVESD	ECHO		[52]M, [52]F	[54], [59]
LVEDV	ECHO		[52]M, [52]F	[53], [54]
LVESV	ECHO		[52]M, [53], [54]	[52]F
EF	ECHO			[52]M, [52]F, [53], [54], [59]
E	ECHO			[54]
A	ECHO			[54]
E/A	ECHO	[52]M		[52]F, [54], [59]
DT	ECHO			[52]M, [52]F
E’	ECHO	[52]M		[52]F, [54]
E/E’	ECHO		[52]M, [59]	[52]F, [54]
LAD	ECHO, cMRI			[54], [56], [59], [63]
AoR	ECHO			[54], [59]
RVD	ECHO			[54]
GLS	STE			[59]
GCS	STE			[59]

ECHO—echocardiography; cMRI—cardiac magnetic resonance imaging; STE—speckle tracking echocardiography; ERP+—subject with early repolarization pattern; LVM—left ventricular mass; IVS—interventricular septum; LVPWT—left ventricular posterior wall thickness; RWT—relative wall thickness; LVEDD—left ventricular end-diastolic diameter; LVESD—left ventricular end-systolic diameter; LVEDV—left ventricular end-diastolic volume; LVESV—left ventricular end-systolic volume; EF—ejection fraction; E—peak early diastolic transmitral flow velocity; A—peak late diastolic transmitral flow velocity; DT—deceleration time; E’—peak velocity of early diastolic mitral annular motion; LAD—left atrial diameter; AoR—aortic root dimension; RVD—right ventricle diameter; GLS—global longitudinal strain; GCS—global circumferential strain.

## Data Availability

Not applicable.
